# The structure of the SOLE element of *oskar* mRNA

**DOI:** 10.1261/rna.049601.115

**Published:** 2015-08

**Authors:** Bernd Simon, Pawel Masiewicz, Anne Ephrussi, Teresa Carlomagno

**Affiliations:** 1 Structural and Computational Biology Unit, European Molecular Biology Laboratory, Heidelberg, D-69117, Germany; 2 Developmental Biology Unit, European Molecular Biology Laboratory, Heidelberg, D-69117, Germany; 3 Helmholtz Zentrum für Infektionsforschung, Braunschweig, D-38124, Germany

**Keywords:** NMR, RNA conformation, mRNA localization, *oskar* mRNA, structural biology

## Abstract

mRNA localization by active transport is a regulated process that requires association of mRNPs with protein motors for transport along either the microtubule or the actin cytoskeleton. *oskar* mRNA localization at the posterior pole of the *Drosophila* oocyte requires a specific mRNA sequence, termed the SOLE, which comprises nucleotides of both exon 1 and exon 2 and is assembled upon splicing. The SOLE folds into a stem–loop structure. Both SOLE RNA and the exon junction complex (EJC) are required for *oskar* mRNA transport along the microtubules by kinesin. The SOLE RNA likely constitutes a recognition element for a yet unknown protein, which either belongs to the EJC or functions as a bridge between the EJC and the mRNA. Here, we determine the solution structure of the SOLE RNA by Nuclear Magnetic Resonance spectroscopy. We show that the SOLE forms a continuous helical structure, including a few noncanonical base pairs, capped by a pentanucleotide loop. The helix displays a widened major groove, which could accommodate a protein partner. In addition, the apical helical segment undergoes complex dynamics, with potential functional significance.

## INTRODUCTION

mRNA localization is a conserved and efficient process that allows confined protein expression and contributes to the functional polarization of cells. This process is important in organismal development, cell migration, and cell fate specification (St Johnston 2005; Besse and Ephrussi 2008; Medioni et al. 2012). In *Drosophila melanogaster, oskar* mRNA localization in the oocyte determines where the abdomen and primordial germ cells will form. *oskar* mRNA transport to the posterior pole requires a polarized microtubule cytoskeleton and its associated motor kinesin (Brendza et al. 2000). It is thought that *trans-*acting factors recognize specific sequences in the *oskar* mRNA transcript and form ribonucleoprotein particles that are competent for kinesin dependent transport (Zimyanin et al. 2008; Ghosh et al. 2014).

**FIGURE 1.**
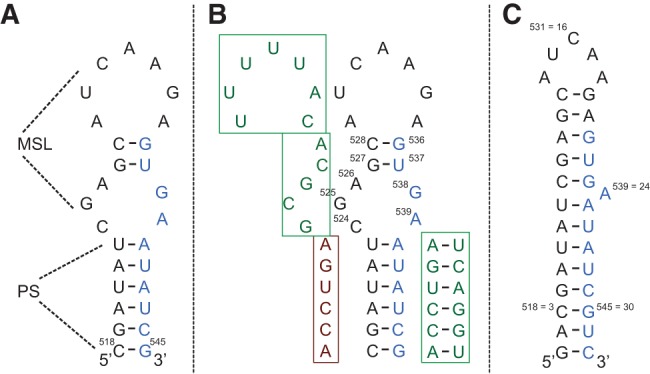
(*A*) Sequence of the SOLE RNA that was identified in Ghosh et al. (2012) as essential for *oskar* mRNA localization. The secondary structure is shown as predicted in Ghosh et al. (2012). The nucleotides in black and blue belong to the first and second exon, respectively. (PS) proximal stem; (MSL) medial stem–loop. (*B*) Summary of the mutant analysis performed in Ghosh et al. (2012). The four mutant sequences are shown in the boxes: Three of them support correct localization (green boxes), while one does not (dark red box). (*C*) Construct of the SOLE RNA used in this study together with the experimentally derived secondary structure. Two base pairs were added at the termini in comparison to the sequence of *A*. The new nucleotide numbering from 1 to 32 is shown with respect to the numbering of *A*. A continuous stacking of base pairs is seen from nucleotides 1–13 and from nucleotides 19–32. A24 is either bulged out or stacked between G23 and A25.

The four core components of the exon junction complex (EJC), a protein complex that is deposited on the mRNA concomitant with splicing 20–24 nucleotides (nt) upstream of exon–exon junctions (Le Hir et al. 2000; Tange et al. 2004), have been found to be required for localization of *oskar* mRNA at the posterior pole (Newmark and Boswell 1994; Hachet and Ephrussi 2001; Mohr et al. 2001; van Eeden et al. 2001; Palacios et al. 2004). Consistent with the notion that the EJC requires splicing for deposition, *oskar* mRNA splicing is required for its localization (Hachet and Ephrussi 2004). Later work identified the formation of a stem–loop structure upon splicing of the first intron. This structure, the SOLE element (Fig. 1), is essential for localization (Ghosh et al. 2012).

The SOLE RNA consists of 18 nt from exon 1 and 10 nt from exon 2, ligated together at the first exon junction site. In vivo mutational analysis established the relevance of the short proximal stem (PS, 6 bp) for localization, suggesting that this structural element participates in the recognition of *trans*-acting factors (Fig. 1B; Ghosh et al. 2012). In contrast, the nucleotide identity in the PS seemed to be unimportant. Nucleotides 524–539 were predicted to fold in the medial stem–loop element (MSL); mutational analysis, designed on the assumption of the MSL structure of Figure 1A, appeared to indicate that this part of the RNA is not essential for function (Ghosh et al. 2012). However, this region can form secondary structures alternative to that in Figure 1A, which might impinge on the design and interpretation of the mutational analysis.

The SOLE RNA sequence is not sufficient for localization. When the SOLE RNA is constitutively present on an *oskar* mRNA transcript, not requiring splicing for its formation, the mRNA is mislocalized (Ghosh et al. 2012). Conversely, mRNA loaded with the EJC but lacking the SOLE sequence is also mislocalized (Ghosh et al. 2012). These facts strongly indicate that the SOLE RNA and the EJC work together to enable *oskar* mRNA localization. It is not known whether there is a direct interaction between the SOLE RNA and the EJC, or a third factor is necessary to connect the two elements.

In the absence of a validated binding partner for the SOLE RNA, we set out to solve its solution structure, with the goal of identifying structural elements that might be essential for protein recognition. We find that at 34°C no internal loop is formed after U523 and before A540; instead, the proximal stem is elongated by five additional base pairs, comprising three noncanonical ones. The long stem presents a widened major groove, which might be key to protein recognition.

## RESULTS

### NMR analysis

The SOLE RNA was produced by in vitro transcription using T7 RNA polymerase and DNA template. A longer RNA was synthesized and cut with a hammerhead ribozyme added in *trans*, to yield the sequence of Figure 1C with a defined 3′-terminus. The final sequence, which was optimized for the activity of the T7 RNA polymerase and of the hammerhead ribozyme, contained two additional base pairs at the termini.

**FIGURE 2.**
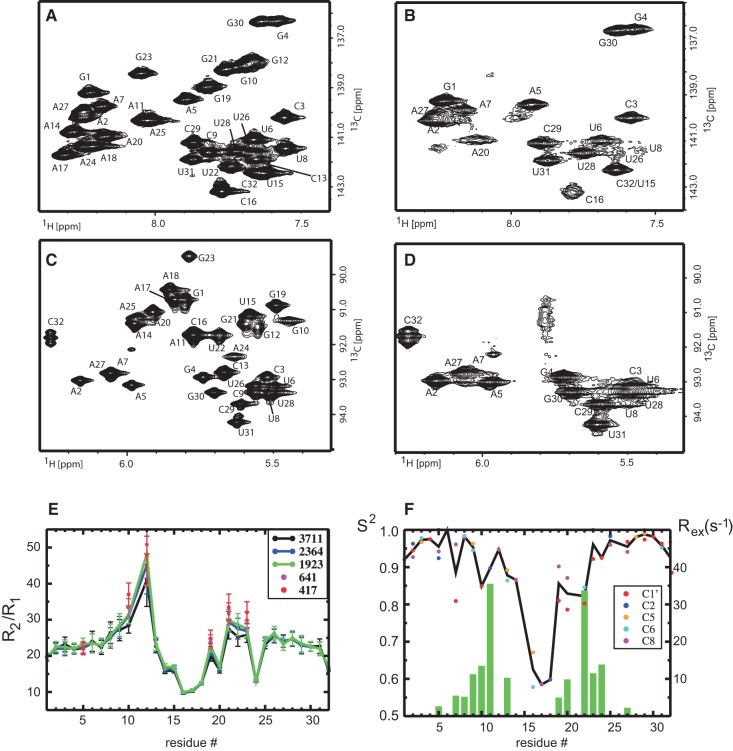
(*A*,*C*) ^13^C–^1^H correlation of the base (*A*) and ribose (*C*) region of the SOLE RNA at 308 K. All resonances are visible in the spectra. (*B*,*D*) ^13^C–^1^H correlation of the base (*B*) and ribose (*D*) region of the SOLE RNA at 283 K. Only the resonances of the first 5–6 bp from the termini are visible. All other resonances are exchange-broadened beyond detection. (*E*) Ratio of transverse and longitudinal relaxation rates of C6/C8 atoms in dependence of the radio frequency field strength *B*_1_ of the *R*_1ρ_ spinlock pulse (given in Hertz in the *inset*). For the two smallest B1 fields, only residues with resonance offsets smaller than 76 Hz are shown. (*F*) Order parameters S^2^ (dots) and exchange contributions *R*_ex_ (bars) characterizing the internal motions of the RNA. The green bars show the *R*_ex_ contribution averaged over all carbon atoms of the corresponding residue. The order parameters are indicated by colored dots, differentiating the different carbon atoms. The black line connects the S^2^ values averaged of all carbons of each residue.

The ^13^C–^1^H correlations of the base and ribose regions acquired at 283 and 308 K show an interesting dynamic behavior of the SOLE RNA (Fig. 2). At 308 K all expected resonances are present in both spectra; at 283 K the resonances of G10, G12, G19, G21, and G23, A11, A14, A17, A18, A24, and A25, U8, and C9 broaden beyond detection. This fact suggests the presence of conformational equilibrium in the intermediate time-scale regime (microseconds–milliseconds) affecting residues 10–25, with exchange rates and populations of the two (or more) folds being temperature dependent. The resonances gradually broaden upon cooling; some of the base resonances move to higher fields, where C6 and C8 atoms belonging to helical structured regions are located. Therefore, it is reasonable to assume that one of the conformations adopted by the MSL element represents a helical structure. Here, we conducted the conformational analysis at 308 K, where we can observe all NMR resonances. Interestingly, this temperature is close to the optimal growth temperature of *D. melanogaster* of 301 K.

To gain more understanding of the dynamic properties of the MSL region, we measured relaxation parameters. *R*_2_/*R*_1_ ratios of the C6 and C8 atoms (Fig. 2E) are almost constant for nucleotides 2–6 and 25–31 (Fig. 1A), indicating a stable structure in this region. Ratios of residues 8–13 and 21–23 are higher than average; in addition, if *R*_1ρ_ is measured at lower *B*_1_ spinlock field strength for residues 10,12,19, 21, 22, and 23, the *R*_2_/*R*_1_ ratios increase further, suggesting conformational exchange in the intermediate time scale (microseconds–milliseconds). Conversely, the *R*_2_/*R*_1_ ratios of residues 14–20 and 24 are lower than average, which demonstrates fast (picoseconds) internal dynamics. All in all, the relaxation parameters are indicative of a complex internal dynamics in the MSL region.

**FIGURE 3.**
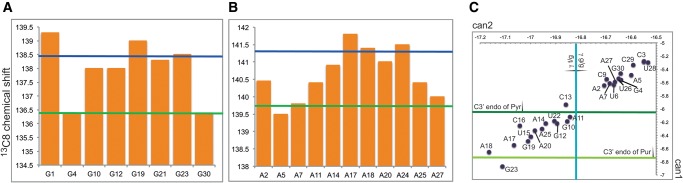
(*A*,*B*) Chemical shifts of the ^13^C8 of G's (*A*) and A's (*B*). The green and blue lines indicate the average chemical shifts in helical and disordered regions, respectively, as defined in Fares et al. (2007). (*C*) Plot of the can1 and can2 coordinates for the riboses of the SOLE RNA (Ohlenschlager et al. 2008). Green lines indicate the minimum can1 for riboses of pyrimidines (dark green) and purines (light green) in the C3′ endo conformation. The blue line indicates the minimum can2 compatible with a g/g conformation of the γ dihedral angle.

Analysis of the chemical shifts of both base and ribose carbons and protons (Fig. 3) reveals clear trends in the secondary structure (Fares et al. 2007). C8 shifts of G4 and G30, as well as A5, A7, and A27 indicate that these nucleotides belong to regular helical structure (Fig. 3A). Conversely, the C8 resonances of G10, G12, G19, G21, and G23 as well as A11, A14, A20, and A25 are shifted to low field, albeit not as much as expected for bulge or disordered regions (2.0 and 1.5 ppm for G and A, respectively). Their moderate low-field shift suggests that they might be “partially” involved in helical structure. Conversely, the C8 chemical shifts of A17, A18, and A24 indicate that they belong to unstructured regions (Fig. 3B). Finally, analysis of U– and C–C6 shifts reveals that U15 and C16 are also disordered. All in all, the chemical shifts suggest that the apical loop encompasses residues 15–18 and that A24 is part of a bulge. The same trends are confirmed by the C1′ frequencies (data not shown) and are in perfect agreement with the relaxation parameters.

The presence of 2 G-N1 and 4 U-N3 imino protons, as well as hydrogen bond mediated G-HN1/C-N3 or U-HN3/A-N1 correlations confirmed the presence of a stable stem for nucleotides (nt) 1–7 and 26–32 (Supplemental Fig. S1).

Despite the absence of slow-exchanging imino protons for the stretch 9–23, the NOEs suggested a stem-like structure for nucleotides 8–13 and 19–23. Strong H6/H8(*i* + 1) − H2′(i) NOEs, accompanied by weak H6/H8(*i* + 1) − H1′(i) NOEs were detected in these regions, as well as cross-strand NOEs between A20-H2 and C13-H1′, A11-H2 and U22-H1′, A24-H2 and C9-H1′. The A20-H2−C13-H1′ and A11-H2−U22-H1′ NOEs indicate base-pairing between G10−U22, A11−G21, G12−A20 and C13−G19. Therefore, in the structure calculation base-pairing was imposed between U8–A25, C9–G23, G10−U22, A11−G21, G12−A20 and C13−G19. The G10–U22 base pair was assumed to be cWW (*cis* Watson–Crick/Watson–Crick) (Leontis et al. 2002), as this is by far the most common in helical elements. The high temperature of the measurement (308 K) and the relaxation data, which indicate the presence of alternative (probably open) conformations for the region 8–13 and 19–23, rationalize the fast exchange of the imino-protons of G10 and U22 with the solvent and consequently their absence in the NMR spectra. For the A11−G21 and G12−A20 base pairs the two most common A−G geometries were considered, which can be accommodated in a helical stretch: tHS (*trans* Hoogsteen/Sugar-edge) and cWW (Leontis et al. 2002). The tHS geometry can be detected in a tailored HNN experiment involving nonexchangeable protons (Leontis et al. 2002). The spectrum showed no correlations for the SOLE RNA, excluding the presence of A–G tHS base pairs. Therefore, the two A−G base pairs were assigned to the cWW geometry.

Next we applied chemical shift analysis of the ribose carbons to determine the ribose pucker and the exocyclic angle γ (Fig. 3C; Ebrahimi et al. 2001; Ohlenschlager et al. 2008) All nucleotides of the lower helical stem have values in the range expected for regular helices (ribose in the C3′ endo conformation and γ angle in the gauche/gauche conformation). The nucleotides in the region 10–22 have the ribose in the C3′ endo conformation, while the γ angles may be in the gauche/trans conformation. In agreement with the analysis of the bases chemical shifts, the nucleotides that deviate most from the helical values are in the region 15–19, where possibly the apical loop is localized. Large deviations from helical values are detected also for G23, supporting the presence of an internal bulge close to this position. ^3^J_H1′,H2′_ coupling constants, measured in an HCCH-E.COSY spectrum, displayed values >4 Hz for A14 and G23, which is indicative of conformational equilibrium between the C3′-endo and C2′-endo ribose conformations.

### Three-dimensional structure of the SOLE RNA in solution

**FIGURE 4.**
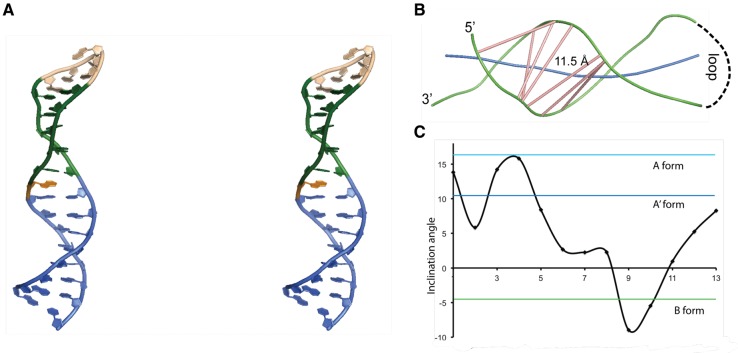
(*A*) Stereo view of a representative structure from the bundle of 10 lowest energy structures of the SOLE RNA (5a17.pdb). Blue, PS stem comprising nucleotides 1–8 and 25–32; green, base pairs in the MSL region, comprising nucleotides 9–13 and 19–23; pink, loop; orange, A24. (*B*) Representation of the major groove width of the SOLE RNA. Green, backbone splines; blue, RNA axis; pink, major groove vectors. The groove width is measured between the spline curves running through the phosphorus atoms. This width is reduced by 5.8 Å to account for the width of the phosphorus backbone. The figure was generated with the program CURVES+. (*C*) Base pair inclination angle with respect to the stem axis at each nucleotide position.

The structure was calculated using 769 NOEs, 206 dihedral angles, and 76 residual dipolar couplings (RDC) restraints (30 for the ribose and 46 for the base C–H vectors). The 10 lowest energy structures converged to a precision of 0.7 Å all heavy atom root mean square deviation (RMSD) (Table 1; Supplemental Fig. S2, PDB entries 5a17 and 5a18, BMRB entries r5a17mr and r5a18mr). A representative structure is shown in Figure 4. The SOLE RNA forms a continuous helical structure comprising nucleotides 1–13, 19–23, and 25–32. A24 is unpaired and is either stacked below A25 or placed in the minor groove. The apical loop is formed by nucleotides 14–18 (AUCAA) and has no well-defined structure. A14 and A18 can potentially form a base pair, whose presence could however not be confirmed by cross-strand NOEs involving any of the A-H2 protons.

**TABLE 1.**
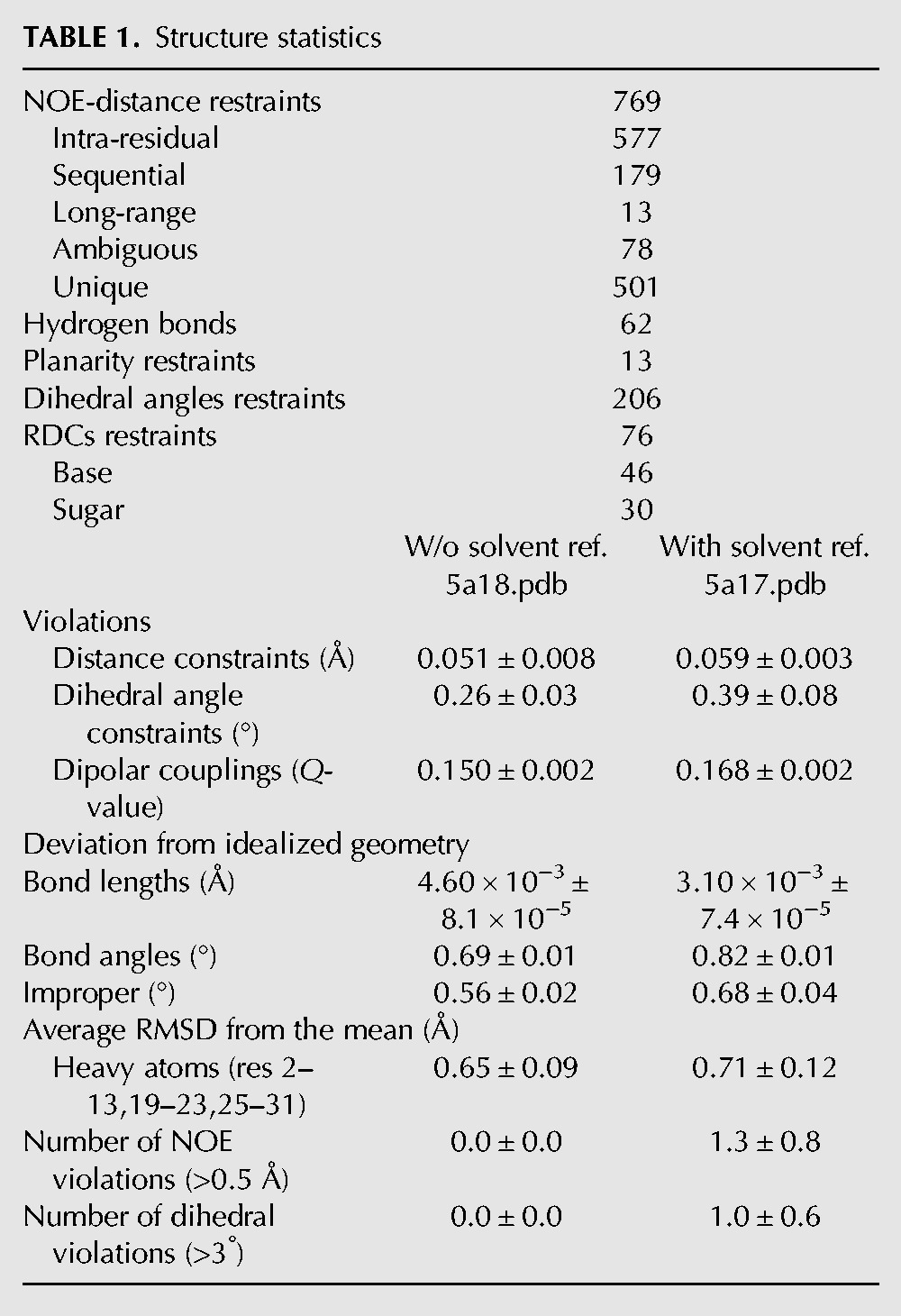
Structure statistics

The lower part of the RNA, from base pair 1–32 to 8–25 (PS), shows helical geometry with a widened major groove (Fig. 4B). Inclination angles of steps 5–28 to 8–25 are between those of B-DNA and A′-RNA, while the major groove width of ∼11 Å is as large as that of B-DNA (Tanaka et al. 1999). The medial stem–loop element (MSL) forms a continuous stack on the PS. Nucleotide stretches 9–13 and 19–23 form two canonical C−G base pairs and three noncanonical G−U and A−G base pairs. Here, the stem geometry deviates substantially from that of A-form helix with inclination angles of steps 9–23 and 10–22 similar to those of B-DNA (Fig. 4C).

In summary, the stretches 3–13 and 19–30 form a continuous helix with a progressively widening major groove that offers a large interaction surface to proteins or peptides.

### Dynamics of the SOLE RNA

The 3D structure allows for a more detailed interpretation of the ^13^C relaxation rates. Here, we used the program ROTDIF (Berlin et al. 2013) to analyze our ^13^C relaxation data (R_1_, R_2_ and heteronuclear NOE), measured at a magnetic field of 14.1 T (150.9-MHz carbon frequency), and model both the diffusion tensor and the parameters of internal dynamics. We performed the analysis with two sets of data, comprising either all residues or only the PS stem. Both analyses resulted in an axially symmetric tensor (Supplemental Table S1), with average correlation time τ_C_ reducing from 6.58 ± 0.11 ns, when fitting the PS stem residues only, to 6.34 ± 0.13 ns with all residues included. This result reflects the dynamic nature of the MSL element. The τ_c_ value is in excellent agreement with the predicted one, after correcting for the higher viscosity of the D_2_O buffer used in our relaxation study (Hardy and Cottington 1949; Scheurer et al. 1999; de la Torre et al. 2000). Within the error bounds, the orientation of the diffusion tensor coincides with the diffusion tensor predicted from the structure, the inertia tensor and the orientation of the RDC alignment frame, as expected in the case of steric (Almond and Axelsen 2002) and electrostatic (Wu et al. 2006) alignment mechanisms, and thus validating the structure (Supplemental Fig. S3).

The parameters characterizing the internal mobility of the SOLE element are shown in Figure 2F. The fast internal motions can be characterized by an order parameter *S*^2^ and an internal correlation time τ_i_, while motions slower than the overall tumbling of the molecule lead to an additional exchange contribution *R*_ex_ to the transverse relaxation rate (Fushman 2012). High-order parameters are observed for residues 1–9 and 23–32, confirming the rigid nature of the PS stem. On the other hand, the three central loop residues U15, C16, and A17 show reduced *S*^2^, which reveal a significant amount of fast dynamics (picosecond time scale). Additional chemical exchange contributions *R*_ex_ are required to fit the relaxation rates of at least one of the carbons in the region 9–13 and 19–24, in agreement with the line broadening observed for these residues in the 2D ^13^C–^1^H correlations (Fig. 2). Thus, it can be concluded that the stem of the MSL region is in conformational exchange with a second minor conformation.

## DISCUSSION

In vivo experiments have demonstrated the importance of splicing at the first exon–exon junction for localization of the *oskar* mRNA transcript at the posterior of the oocyte. Splicing results in two events: (1) formation of the SOLE, a 28-nt RNA sequence, which consists of 18 nt of exon 1 and 10 nt of exon 2, and (2) deposition of the EJC complex 20–24 nt upstream of the exon–exon junction. Both events are required for the correct localization of the *oskar* mRNA transcript (Ghosh et al. 2012). This has led to the hypothesis that the SOLE RNA interacts, either directly or with the help of adaptor proteins, with the EJC. One possibility is that the EJC–SOLE RNA complex is then coupled to the kinesin motor for transport.

RNA recognition by proteins occurs mainly in the minor groove or at nonhelical elements. The deep and narrow shape of the A-form RNA major groove does not allow interactions with either peptides or proteins. However, in the past years several structures were solved that show RNA helices in a different geometry, with a widened major groove (Tanaka et al. 1999; Bullock et al. 2010; Asami et al. 2013). The so called A′-form helix can be achieved with nearly no changes in RNA backbone angles and displays an unwound helix and a major groove almost as wide as that of B-DNA (Tanaka et al. 1999). In a very elegant study (Bullock et al. 2010), the Lukavsky laboratory demonstrated that the 44-nt long *D. melanogaster* K10 transport and localization signal (TLS), which acts in *K10* mRNA transport by the dynein motor, displays A′-form conformation. In the *K10* RNA two spatially registered widened major grooves seem to be required for transport.

Reminiscent of the K10 TLS, the SOLE RNA displays a stem with a widened major groove. The stem structure is essential for transport, as disruption of base pairs in the PS region leads to mislocalized *oskar* transcripts (Ghosh et al. 2012); the phenotype can be rescued by reestablishment of the base-pairing, in a non-sequence-specific manner. Thus, it is reasonable to hypothesize that cofactor recognition takes place through the widened major groove of the stem.

A few examples of protein–RNA major groove recognition exist in the literature, which involve A′-form helical conformation. The ROQ domain of the protein Roquin binds the TNF-α (tumor necrosis factor-α) CDE (constitutive decay element) stem–loop RNA facing its major groove and interacting with the apical part of the stem, the loop and the backbone on one side of the helix (Schlundt et al. 2014). The CDE RNA stem adopts an A′-form conformation, as predicted by the presence of a continuous stack of >3 purines in one strand. Similarly, The boxB stem–loop RNA of both the bacteriophage λ and ϕ21 recognizes a distorted helix of the respective N-proteins in the major groove (Legault et al. 1998; Cilley and Williamson 2003). As for Roquin, the N protein contacts mainly the loop, the apical part of the stem and the backbone on one side of the boxB RNA helix. Both boxB stems feature stacks of >3 consecutive purines, which lead to A′-form conformation.

In the SOLE element, the RNA helix extends from the PS to the MSL region, where the major groove is widened further by the presence of two purine–purine base pairs (Fig. 1). This geometry was unexpected based on structure prediction (Fig. 1A), which suggested the presence of a large internal loop, a very short stem and a 7-nt apical loop. The experimental structure of the SOLE RNA shows no internal loop and continuous stacking of five additional base pairs on the PS stem. Interestingly, the sequence of nucleotides 524–528 was mutated without effect on transport (Fig. 1B; Ghosh et al. 2012), suggesting that this region does not need to assume a specific conformation to sustain function. However, the mutant sequence that was tested can, like the wild-type sequence, support formation of five base pairs (^524^G−^539^A, ^525^C−^538^G, ^526^G−^537^U, ^527^C−^536^G, ^528^A−^535^A), which would stack on the PS stem without interruption. The elongation of the stem could serve the purpose of stabilizing the unwound helical structure of the PS region; alternatively, it could provide an additional interaction surface to cofactors.

As for the K10 TLS, SOLE-dependent mRNA localization does not require a specific nucleotide sequence, but rather depends on RNA secondary structure. However, the structural details and the pattern of intermolecular recognition seem to diverge for the K10 TLS and the SOLE RNAs. Localization of *K10* mRNA necessitates two spatially registered widened major grooves: In the K10 TLS, A′-form helices are induced in the upper and lower parts of a long stem by a continuous stack of >3 purines in one strand. In contrast, the sequence of the SOLE RNA lacks a continuous purine stack in the PS stem and does not suggest the formation of A′-form helix. The unwinding of the helix from A- to A′-form starts only at the center of the PS stem and is consolidated by the purine stacking of the noncanonical base pairs in the MSL region. In addition, the short dimension of the SOLE element does not support the formation of two spatially registered widened major grooves.

Three noncanonical base pairs are the distinctive feature of the SOLE RNA in the MSL region. Non-WC base pairs as part of a stem have been long recognized to widen the major groove and serve as platforms for protein recognition (Hermann and Westhof 1999). For example, a *cis* WW A–G base pair in the HIV RRE (Rev response element) RNA stem functions as recognition site for the Rev peptide helix, which deeply penetrates the widened RNA major groove (Battiste et al. 1996).

The importance of the MSL nucleotides of the SOLE element is suggested by their conservation in all *Drosophila* species (Supplemental Fig. S4A). The identity of the base pair ^524^C−^538^G, as well as the bulged out ^539^A, is consistently preserved; the next ^525^G−^537^U base pair can be formed in all but three species (*D. virilis, grimshawi*, and *willistoni)*, while the noncanonical ^526^A−^536^G base pair is conserved either as such or as the isosteric A–A base pair (Supplemental Fig. S4). The ^527^G−^535^A base pair is present in all but two species (*D. pseudoobscura* and *D. persimilis*) as G–A or A–A, while the last base pair of the stem is less consistent. Most important, the disordered AUCAA loop is conserved in all species, suggesting that these exposed nucleotides might build a recognition element for proteins or nucleic acids. Nevertheless, its mutation to UUUUU does not affect localization (Ghosh et al. 2012), raising the hypothesis that the SOLE structural motif may exert multiple functions. All in all, the elongated helix is compatible with the sequence of most *Drosophila* SOLE elements and might be induced by the favorable purine stacking energy.

The structure of the SOLE RNA solved here allows us to revisit the structure predictions of putative SOLE elements in other organisms (Supplemental Fig. S4B; Lynch et al. 2011; Ghosh et al. 2012). Despite the presence of several Oskar orthologs (Lynch et al. 2011), both cDNA and genomic information is available only for four organisms other than *Drosophila*. The *Anopheles gambiae* SOLE element between position −18 of exon 1 and +10 of exon 2 can fold in a 11-bp helix, which includes five noncanonical G–U, A–G and A–A base pairs and is capped by a hexanucleotide loop (Supplemental Fig. S4). In *Culex quinquefasciatus* a 10-bp helix can form between position −16 of exon 1 and +10 of exon 2; the helix comprises four noncanonical G–U, A–G, and A–A base pairs and is capped by a pentanucleotide loop. In *A. aegypti*, a continuous stack of 10 bp, comprising six noncanonical G–U, G–A, A–A and possibly C–U pairs, is capped by a trinucleotide loop. The SOLE elements of the three organisms share common structural features: (1) The helix is 10–11 steps long and starts between position −18 and −15 of exon 1; (2) the apical part of the helix contains two purine–purine base pairs, which consistently widen the major groove; (3) the helix is poor in G–C content, suggesting a dynamic equilibrium between different structures. Interestingly, the loop is consistently rich in A's and U's, but its length is not conserved. Conversely, the SOLE element of *Nasonia vitripennis* is predicted to fold in a stem–loop structure with a few different features: The stem is only 8-bp long and contains one purine–purine base pair in the basal rather than in the apical part; however, similar to the *Drosophila* SOLE stem, an A′-form helical structure with a widened major groove is predicted for the stem of *N. vitripennis*, due to the continuous stack of three or more purines on one strand. With 10 nt, the apical loop is the longest among all species.

It remains an open question, whether the dynamic behavior of the MSL element of the SOLE RNA could have a temperature-dependent regulation function. Additional in vivo experiments, which probe the activity of mutant SOLE elements with altered structural dynamics, are needed to answer these questions. It is tempting to speculate that melting of the MSL region might be needed to hand over the *oskar* mRNA to different protein partners during transport.

## MATERIALS AND METHODS

### RNA preparation

The RNA 5′-GACGAUAUCGAGCAUCAAGAGUGAAUAUCGUC-3′ was prepared by in vitro transcription using T7-polymerase produced “in-house,” ^13^C/^15^N-labeled (Silantes) or unlabeled (Sigma-Aldrich) rNTPs and plasmid double-stranded DNA templates. The RNA was purified by denaturing 12% polyacrylamide gel electrophoresis. Unlabeled *trans*-acting hammerhead RNA was used to obtain RNA with homogenous 3′ end (Milligan et al. 1987).

NMR samples at 0.3–0.4 mM concentration were prepared by dissolving the RNA in 0.35 mL buffer (20 mM sodium phosphate buffer, pH 6.5). NMR experiments involving exchangeable protons were performed in a H_2_O:D_2_O mixture (9:1). All other experiments were performed in 99.96% D_2_O (Sigma-Aldrich). For residual dipolar coupling (RDC) experiments, 10–15 mg/mL Pf1 phages (ASLA Biotech) were added, resulting in a splitting of the deuterium solvent line of ∼13 Hz.

### NMR spectroscopy

NMR spectra were recorded on Bruker Avance 600 and 800 MHz spectrometers. 2D ^13^C–^1^H correlations of the base and ribose regions revealed that at low temperatures a conformational exchange process causes the disappearance of several resonances between nucleotides 10 and 25. At 308 K all resonances become visible: This temperature was chosen for all following experiments.

The assignment of the RNA resonances relied on 3D (^1^H, ^13^C, ^15^N) H_b_CN_b_ (Fiala et al. 2000), 3D (^1^H, ^13^C, ^15^N) H_s_CN_b_ (Sklenar et al. 1998; Brutscher and Simorre 2001), 3D HCCH-COSY-TOCSY (mixing time 5.4 msec) (Hu et al. 1998), and 3D (^1^H, ^13^C, ^1^H) edited NOESY (150 msec, 200 msec) (Zwahlen et al. 1997). The ribose spin systems were assigned from the 3D HCCH-COSY-TOCSY spectrum; the 3D HCN spectra yielded 70% of the intranucleotide correlations between the H1′ and the H6/H8 protons, which were confirmed from the 3D ^13^C-edited NOESY spectrum. The latter spectrum allowed assigning the other 30% nucleotide spin systems as well as completing sequential assignment. The totality of the ^1^H chemical shift assignments is close to 100% for nonexchangeable protons. Imino protons were assigned from 2D NOESY spectra with 150-msec mixing time. Only imino protons of nucleotides 1–9 and 26–32 were visible in the NOESY at 308 K. To detect ^2^J_NN_ couplings across hydrogen bonds in Watson–Crick and noncanonical G–A base pairs, 2D HNN-COSY spectra were recorded using correlations to both exchangeable and nonexchangeable protons (Dingley and Grzesiek 1998; Hennig and Williamson 2000). Seven signals belonging to the 4 A–U and 3 G–C base pairs of the lower part of the stem were observed at 288 K in the 2D HNN-COSY detecting exchangeable protons (Supplemental Fig. S1). The four A–U base pairs were confirmed in the 2D HNN-COSY detecting nonexchangeable H2 protons. All spectra were analyzed with Felix (FELIX NMR).

Relaxation rates were recorded using proton detected sensitivity enhanced HSQC spectra with a scan-wise interleaved data recording scheme and standard pulse programs to obtain *R*_1_, *R*_1ρ_ and heteronuclear NOE (Yamazaki et al. 1994); a composite pulse ^1^H decoupling scheme was used during the spinlock period (Vallurupalli et al. 2012). The power of the spinlock field was calibrated by a 2D nutation experiment (Guenneugues et al. 1999). Selective carbon pulses were used to achieve selective magnetization transfer during the INEPT delays and refocus carbon–carbon scalar couplings (Hansen and Al-Hashimi 2007). R_1_ and R_1ρ_ experiments used relaxation delays of 20, 60(×2), 100, 200, 400, 700(×2), and 1000 msec and 0, 8, 16(×2), 24, 32, 40, 48, 56, and 64 msec, respectively. To ensure proper alignment of the magnetization along the spinlock field, separate *R*_1ρ_ experiments were recorded for C1′, C2, C5, and C6/C8 resonances, leading to a maximal resonance offset of 980 Hz from the spinlock frequency; only residues within ±76 Hz were evaluated for the lower spinlock field strengths (C8 of residues 10, 12, 19, 21, and 23). Relaxation rates were fit to a mono-exponential decay and errors estimated using a Monte Carlo approach within nmrview (Johnson and Blevins 1994).

The distance restraints were measured from the 3D ^13^C-edited NOESY at 150-msec mixing time. The integration of the NOE volumes and the calibration of the distances were performed by an internal routine of the program Felix. Ribose puckers were determined by evaluation of the ^3^J_H1′–H2′_ coupling from a 3D HCCH-E.COSY spectrum (Schwalbe et al. 1994).

Residual dipolar couplings (RDCs) were calculated as the difference between ^1^H–^13^C couplings measured for isotropic and partially aligned samples. RDCs were obtained for the following internuclear vectors: H8–C8 (Pu), H6–C6 (Py), H5–C5 (Py), H2–C2 (Ade), and C1′–H1′ (ribose). Seventy-six RDCs could be measured (45 in the bases and 31 in the riboses).

### Structure calculations

Structures were calculated using the Aria 1.2/CNS 1.1 set-up (Brunger et al. 1998; Linge et al. 2003). Six hundred ninety-one unambiguous and 78 ambiguous NOE distances were categorized as weak (3.2–6.0 Å), medium (2.4–4.0 Å), or strong (1.4–3.0 Å) (Table 1). The ribose conformation of 28 nt was restrained to the *C3*′*-*endo range by estimating the magnitude of the ^3^J_H1′–H2′_ scalar couplings in the HCCH-E.COSY 3D experiment. Fourteen γ dihedral angles were restrained to the *gauche*^+^ range in the PS stem, as confirmed by analysis of the can2 coordinate (Ohlenschlager et al. 2008). The χ angles of 16 nt were restricted on the basis of the intensities of the intranucleotide H8–H1′ (Pu), and H6/H5–H1′ (Py) NOEs. Loose, nonexperimental restraints to the most populated regions were added for the α (180° ± 150°), β (180° ± 110°), ε (−125° ± 75°), and ζ (180° ± 150°) angles of all nucleotides, excluding loop nucleotides 14–18 and the bulged out A24.

Hydrogen bonds of WC base pairs were detected in HNN correlations and in NOESY experiments for stretch 1–7 and 26–32 (Supplemental Fig. S1). The six base pairs of stretch 8–25 could not be verified experimentally, as the imino protons of this “unstable” part of the stem exchange too rapidly with water at 308 K. However, both the NOEs and the RDCs clearly indicated the formation of a stem with regular base stacking. Therefore, in the final calculations, hydrogen bonds were imposed for U8–A25, C9–G23, G10–U22, A11–G21, G12–A20, and C13–G19. The A–G base pairs were set to be *cis* WC–WC, as this configuration best fitted the NOEs and dipolar coupling data and by exclusion (see Results). During the calculations, hydrogen bonds were maintained by distances restraints, while planarity was enforced through weak planarity restraints (5 kcal mol^−1^ Å^−2^).

One hundred structures were calculated without using the automated assignment or the distance calibration options of Aria 1.2. The simulated annealing (SA) protocol starts with a high-temperature torsion angle simulated annealing phase with 100,000 steps at 20,000 K (time step of 27 fs). This is followed by a torsion angle dynamic cooling phase from 20,000 K to 2000 K in 100,000 steps and by two Cartesian dynamic cooling phases with a time step of 3 fs (from 2000 to 1000 K in 100,000 steps and from 1000 to 50 K in 80,000 steps, respectively).

In a second step, the structures were refined adding RDC data to the structural restraints. The initial values for the rhombic (*r*) and axial (*D*_a_) components of the alignment tensor were obtained by evaluating the RDCs pattern distribution; an intensive grid search was performed around these values for both *D*_*a*_ and *r*, where the dipolar coupling energy term was evaluated as a function of the alignment tensor; the energy profiles revealed a minimum for *D*_a_ = 19.1 and *r* = 0.23 and these values were used in the refinement. The final ensemble of 10 structures (Supplemental Fig. S2) was refined in a shell of water molecules (Linge et al. 2003; Nilges et al. 2008; Nozinovic et al. 2010).

The RDC refined structures showed no NOE (>0.5 Å) or dihedral angle (>3°) violations. The final structures were analyzed using MolMol (Koradi et al. 1996); figures were prepared with PyMOL (http://www.pymol.org).

### Analysis of the relaxation parameters

The relaxation rates *R*_1_, *R*_2_, and heteronuclear NOEs were analyzed using ROTDIF (Berlin et al. 2013). For τ_c_ > 3 ns the relaxation rates are dominated by the low frequency spectral density components J(0) and J(ω_C_). Instead of the *R*_2_/*R*_1_ ratio, ROTDIF uses the related ratio of spectral density components ρ = 4J(0)/3J(ω_C_), which is initially estimated from the relaxation rates and subsequently refined while modeling the diffusion tensor and internal dynamics. The robust least-squares method was used for estimating the experimental diffusion tensor. In small RNA molecules the ^13^C relaxation rates of proton bound carbons are dominated by chemical shift anisotropy (CSA) and the dipolar contribution of the directly attached proton. For an overall tumbling correlation time τ_c_ = 6 ns, the dipolar contributions of neighboring carbons are smaller than 1% (Ferner et al. 2008) and can be neglected. In addition, the program assumes collinearity between the dipolar and CSA interaction tensors. This assumption is not correct for the base carbons: The base shielding tensors are approximately axially symmetric (δ_11_∼δ_33_), but the unique axis δ_22_ is rather perpendicular than collinear to the dipolar interaction tensor (Sitkoff and Case 1998; Stueber and Grant 2002; Hansen and Al-Hashimi 2006). Nonetheless, the program was applied successfully to fit relaxation data of DNA and RNA molecules (Berlin et al. 2013). To avoid the selection of complex motional models that may be caused by these systematic errors, we multiplied the errors for the measured relaxation rates by a factor of three, since the selection of local dynamics is based on statistical arguments.

## SUPPLEMENTAL MATERIAL

Supplemental material is available for this article.

## Supplementary Material

Supplemental Material
