# Supplementary material for: The structure of the SOLE element of oskar mRNA
Source: RNA. 2015 Aug;21(8):1444–53. doi: 10.1261/rna.049601.115 (PMC4509934; doi:10.1261/rna.049601.115)
Supplement: Supplemental Material [file supp_21_8_1444__index.html]

The structure of the SOLE element of oskar mRNA — The structure of the SOLE element of oskar mRNA — Supplemental Material 

# The structure of the SOLE element of *oskar* mRNA

## Supplemental Material

**Files in this Data Supplement:**

- Supp Material.docx
