# Supplementary material for: The structure of the SOLE element of oskar mRNA
Source: RNA. 2015 Aug;21(8):1444–53. doi: 10.1261/rna.049601.115 (PMC4509934; doi:10.1261/rna.049601.115)
Supplement: Supplemental Material [file supp_049601.115_SuppMaterial.docx]

**The structure of the SOLE element of *oskar* mRNA**

Bernd Simon^1^, Pawel Masiewicz^1^, Anne Ephrussi^2^ and Teresa Carlomagno^1,3,*^

^1^ Structural and Computational Biology Unit, European Molecular Biology Laboratory, Heidelberg, D-69117, Germany

^2^ Developmental Biology Unit, European Molecular Biology Laboratory, Heidelberg, D-69117, Germany

^3^ Helmoltz Zentrum für Infektionsforschung, Braunschweig, D-38124, Germany

To whom correspondence should be addressed. Tel: +49 6221 3878552; Email: carlomag@embl.de

**Supplementary Material**

**A**

**
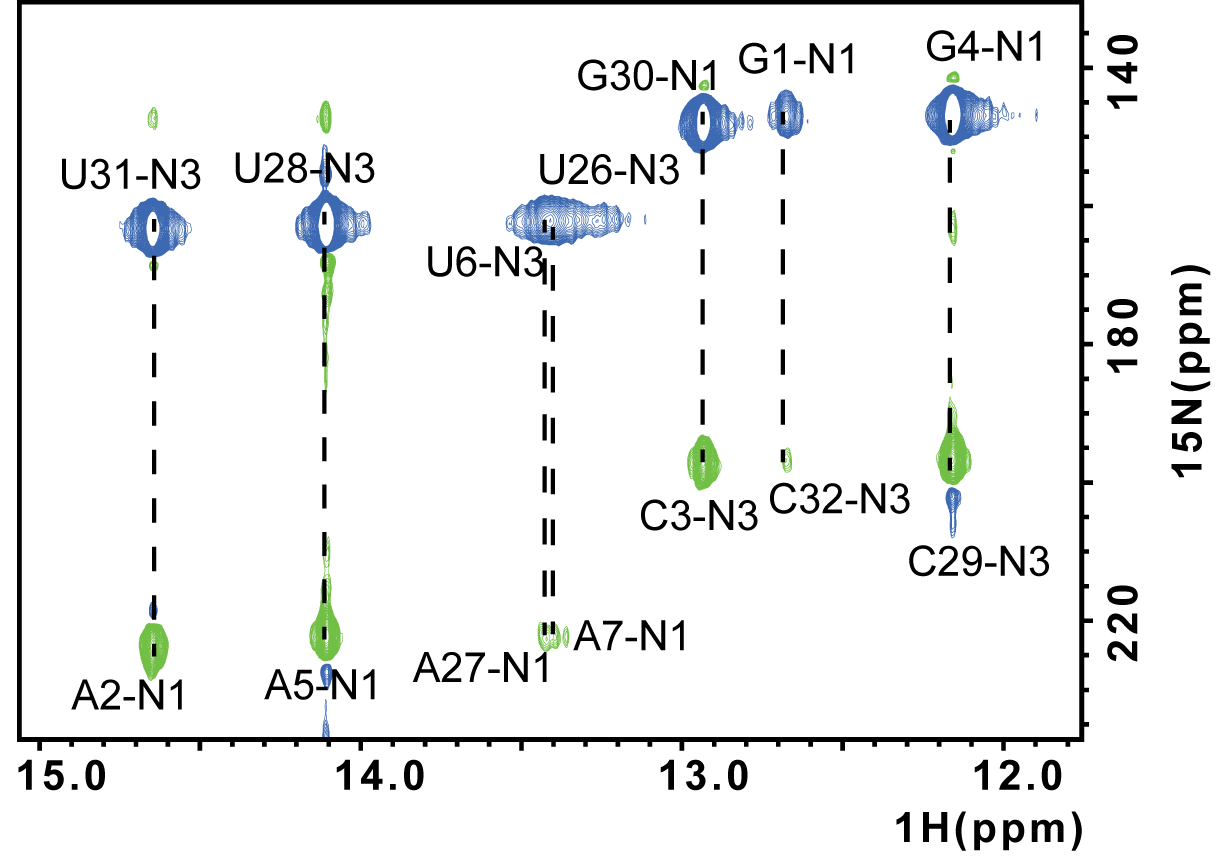
**

**B**

**
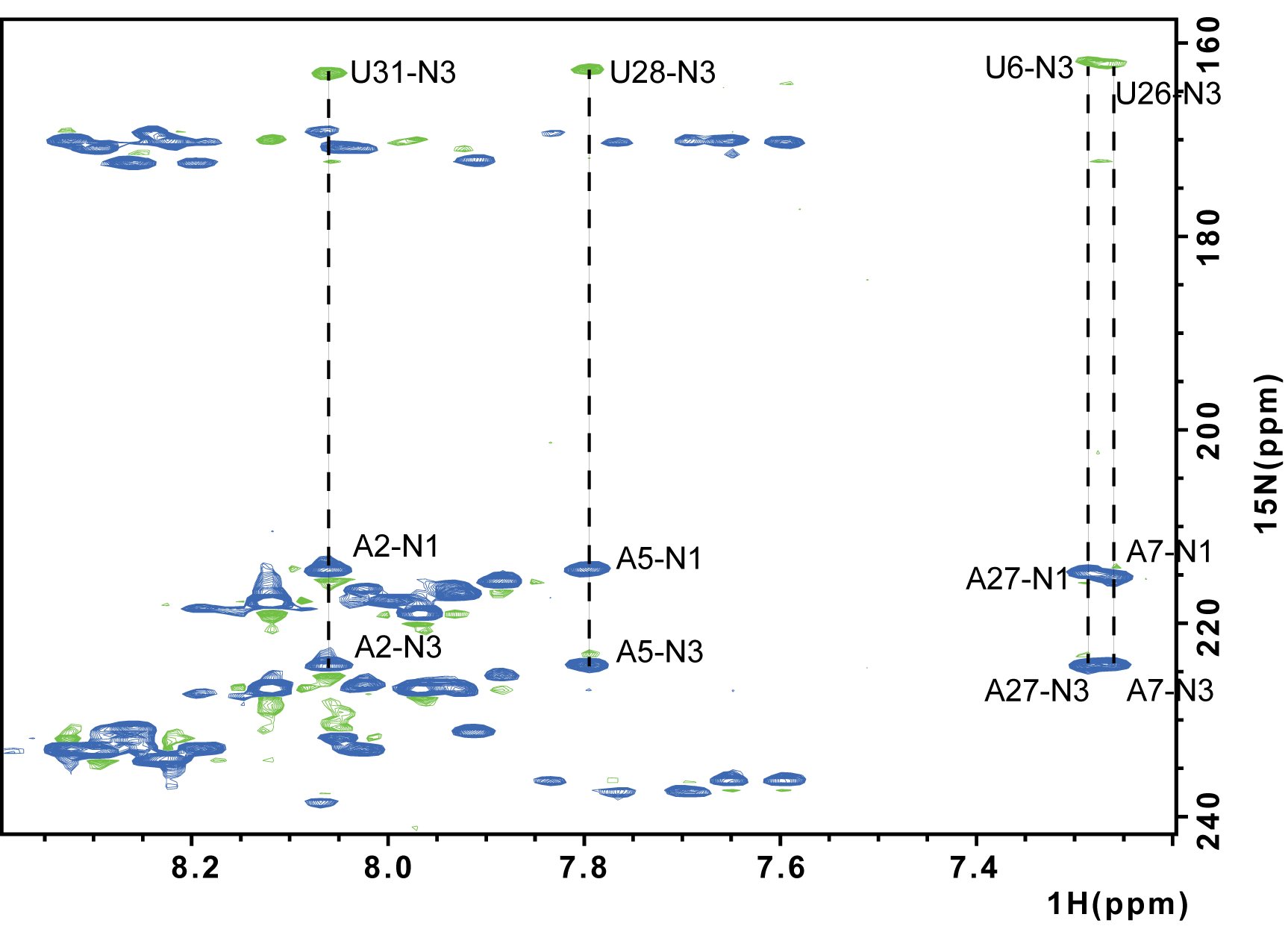
Supplementary Figure 1. A.** 2D HNN-COSY spectrum detecting exchangeable imino protons acquired at 288 K. The base pairs 1-7 and 26-32 are visible. Additional U-imino protons at the ^1^H chemical shift of ca. 13.4 ppm belong to the U8-A25 base pair and possibly to the second conformation of the SOLE RNA. At 308 K the G1, U6 and U26 imino protons are not visible. **B.** 2D HNN-COSY spectrum detecting non-exchangeable base protons acquired at 308 K. Four A-U base pairs of the PS stem (A2-U31, A5-U28, U6-A27 and A7-U26) are detected.


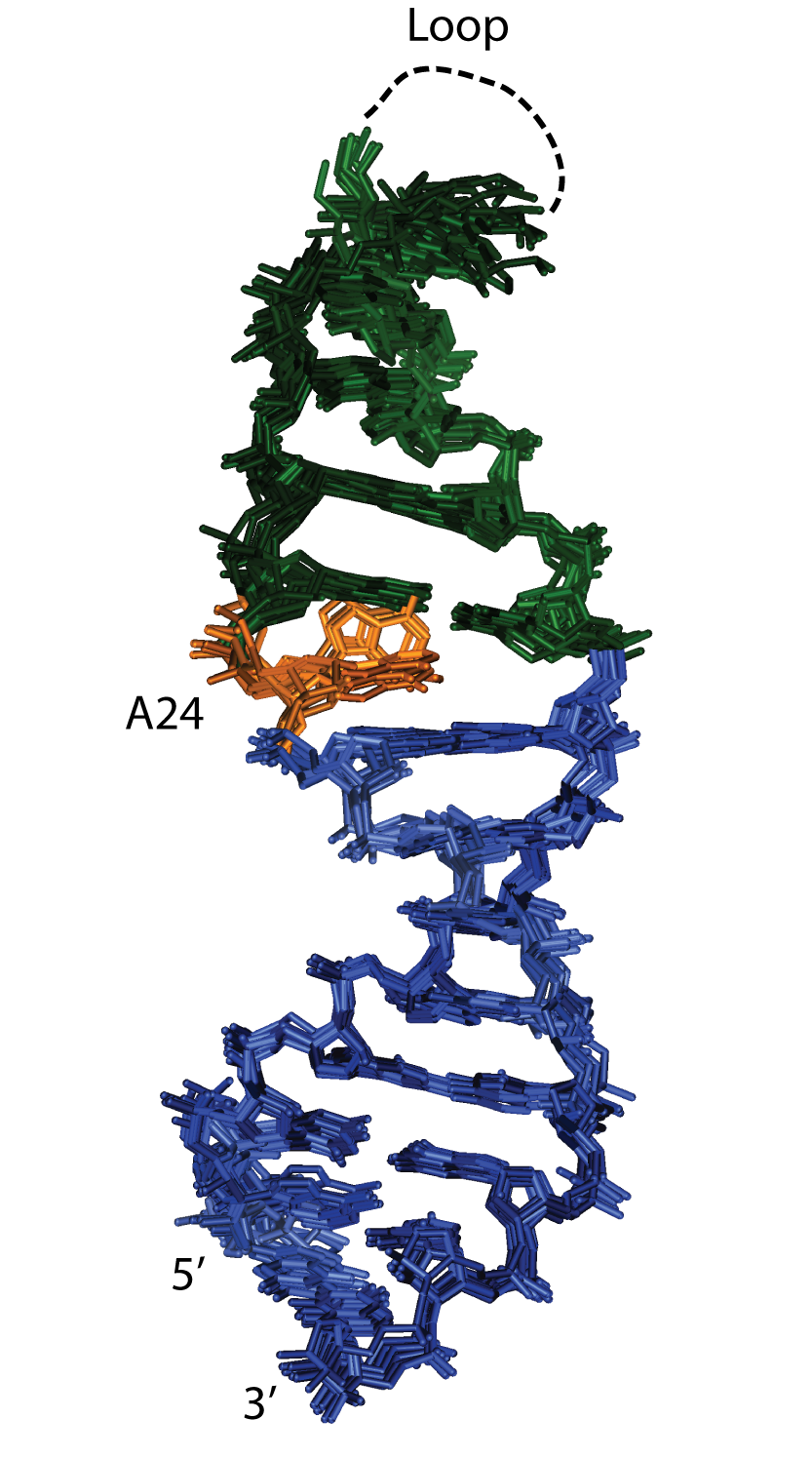


**Supplementary Figure S2. Superposition of the 10 lowest energy structures after refinement in explicit solvent (5a17.pdb).** The rmsd of all heavy atoms of nt 1-13, 19-23 and 25-32 is 0.7 Å. Blue, PS stem comprising nt 1-8 and 25-32; green, base pairs in the MSL region, comprising nt 9-13 and 19-23; orange, A24. The loop residues 14-18 are not shown. A24 is either stacked between G23 and A25 or bulged out.

**
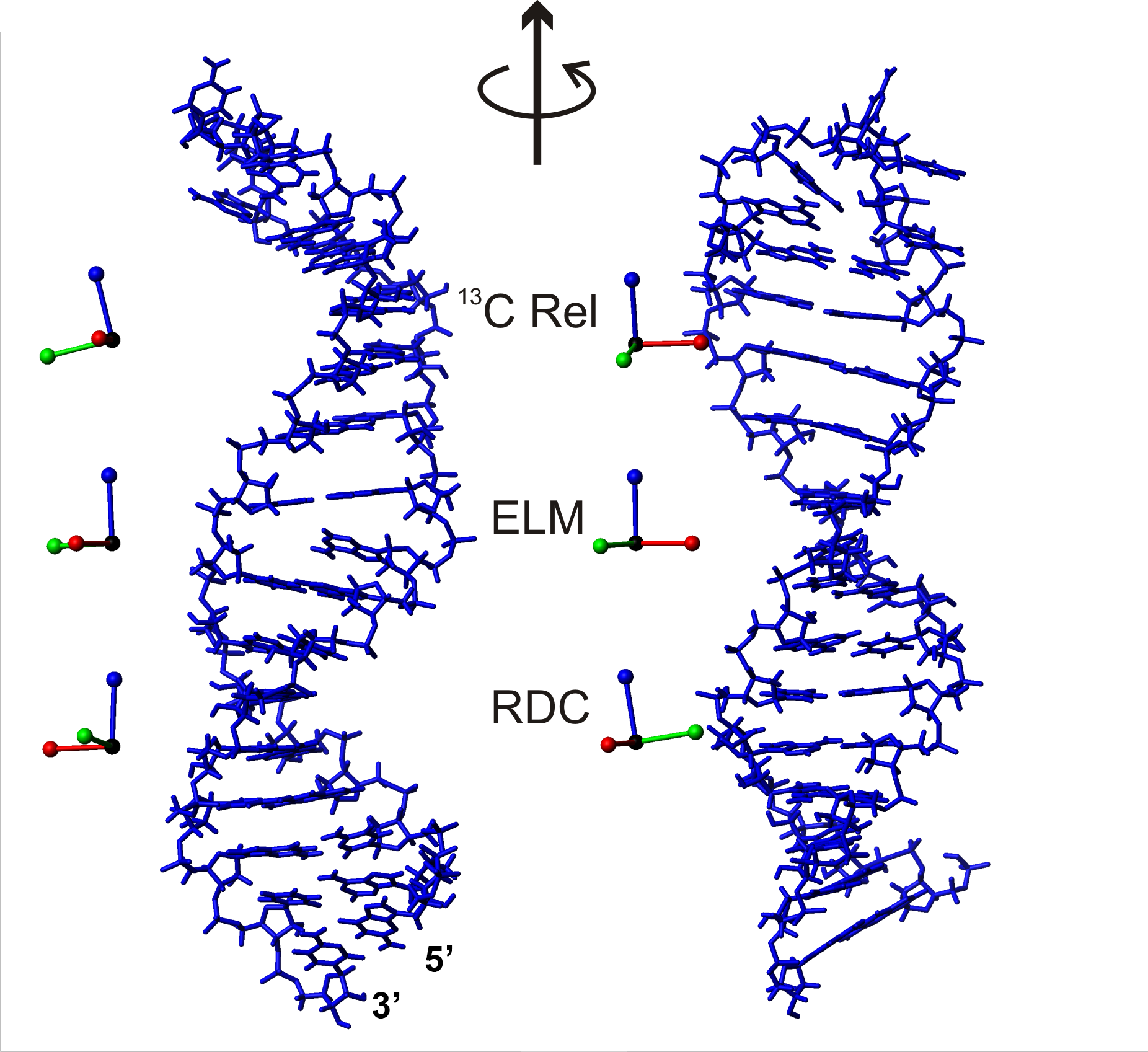
**

**Supplementary Figure S3.** Lowest energy structure with coordinates in the inertia frame. The coordinate frames indicate the orientation of the diffusion tensor fitted to the ^13^C relaxation data (top, ^13^C Rel), the diffusion tensor predicted from the coordinates using the ellipsoid model (middle, ELM) and the RDC alignment frame (bottom), with the origin in black, and the x,y,z axis in red, green and blue respectively. The two views are rotated by 90° around the z-axis of the inertia frame. The x and y axis of the RDC derived tensor are swapped with respect to those of the other two tensors, reflecting the axial symmetry of all tensors.

A

1 10 18 28

| *D. virilis* | **C** | **G** | **A** | **U** | **A** | **U** | **C** | G | **A** | **G** | C | **A** | **U** | **C** | **A** | **A** | A | **A** | **A** | G | **G** | **A** | **A** | **U** | **A** | **U** | **C** | **G** |
| --- | --- | --- | --- | --- | --- | --- | --- | --- | --- | --- | --- | --- | --- | --- | --- | --- | --- | --- | --- | --- | --- | --- | --- | --- | --- | --- | --- | --- |
| *D. grimshawi* | **C** | **G** | **A** | **U** | **A** | **U** | **C** | G | **A** | **G** | C | **A** | **U** | **C** | **A** | **A** | A | **A** | **A** | G | **G** | **A** | **A** | **U** | **A** | **U** | **C** | **G** |
| *D. pseudoobscura* | **C** | **G** | **A** | **U** | **A** | **U** | **C** | **G** | **A** | C | C | **A** | **U** | **C** | **A** | **A** | A | A | **A** | **U** | **G** | **A** | **A** | **U** | **A** | **U** | **C** | **G** |
| *D. persimilis* | **C** | **G** | **A** | **U** | **A** | **U** | **C** | **G** | **A** | C | C | **A** | **U** | **C** | **A** | **A** | A | A | **A** | **U** | **G** | **A** | **A** | **U** | **A** | **U** | **C** | **G** |
| *D. ananassae* | **C** | **G** | **A** | **U** | **A** | **U** | **C** | **G** | **A** | **G** | **U** | **A** | **U** | **C** | **A** | **A** | **G** | **A** | **G** | **U** | **G** | **A** | **A** | **U** | **A** | **U** | **C** | **G** |
| *D. melanogaster* | **C** | **G** | **A** | **U** | **A** | **U** | **C** | **G** | **A** | **G** | **C** | **A** | **U** | **C** | **A** | **A** | **G** | **A** | **G** | **U** | **G** | **A** | **A** | **U** | **A** | **U** | **C** | **G** |
| *D. yakuba* | **C** | **G** | **A** | **U** | **A** | **U** | **C** | **G** | **A** | **A** | **C** | **A** | **U** | **C** | **A** | **A** | **G** | **A** | **G** | **U** | **G** | **A** | **A** | **U** | **A** | **U** | **C** | **G** |
| *D. simulans* | **C** | **G** | **A** | **U** | **A** | **U** | **C** | **G** | **A** | **A** | **C** | **A** | **U** | **C** | **A** | **A** | **G** | **A** | **G** | **U** | **G** | **A** | **A** | **U** | **A** | **U** | **C** | **G** |
| *D. sechelia* | **C** | **G** | **A** | **U** | **A** | **U** | **C** | **G** | **A** | **A** | **C** | **A** | **U** | **C** | **A** | **A** | **G** | **A** | **G** | **U** | **G** | **A** | **A** | **U** | **A** | **U** | **C** | **G** |
| *D. erecta* | **C** | **G** | **A** | **U** | **A** | **U** | **C** | **G** | **A** | **A** | **C** | **A** | **U** | **C** | **A** | **A** | **G** | **A** | **G** | **U** | **G** | **A** | **A** | **U** | **A** | **U** | **C** | **G** |
| *D. willistoni* | **C** | **G** | **A** | **U** | **A** | **U** | **C** | G | **A** | **A** | C | **A** | **U** | **C** | **A** | **A** | A | **A** | **G** | G | **G** | **A** | **A** | **U** | **A** | **U** | **C** | **G** |
| *D. mojavensis* | **C** | **G** | **A** | **U** | **A** | **U** | **C** | **A** | **A** | **G** | C | **A** | **U** | **C** | **A** | **A** | A | **A** | **A** | **U** | **G** | **A** | **G** | **U** | **A** | **U** | **C** | **G** |

B


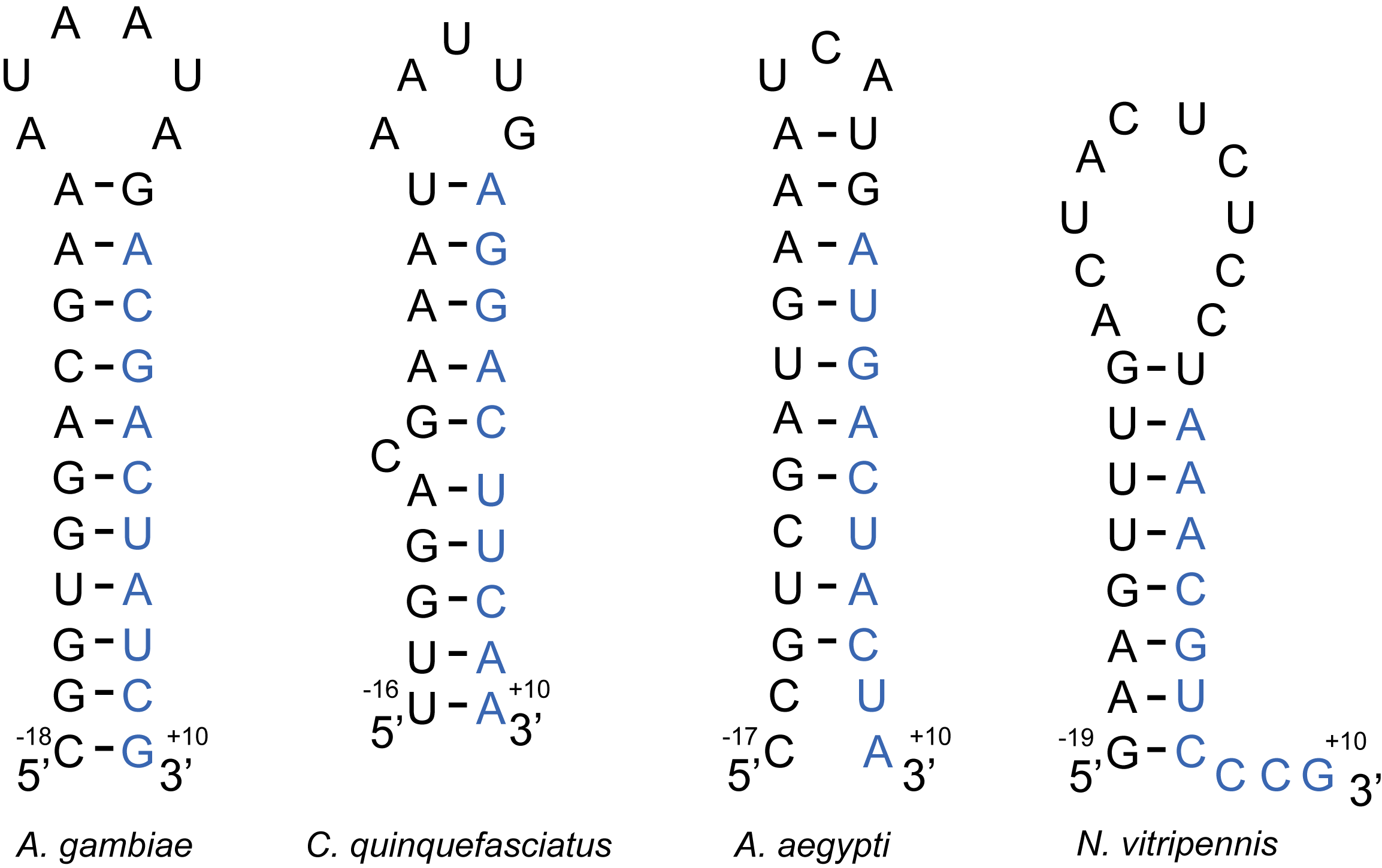


**Supplementary Figure S4. A.** Alignment of SOLE sequences (nt 1-28) from *Drosophila* species. Blue, conserved base-pairs of the PS stem; green, conserved base pairs of the MSL stem; orange, bulged out adenosine; rosa, loop. B. Putative stem-loop structures of SOLE elements from other insects. Black, the last 16-19 nucleotides of exon 1; blue, the first 10 nucleotides of exon 2.

**Supplementary Table 1.** Diffusion tensors computed from the relaxation data.

|  | Dx | Dy | Dz | α | β | γ | τ_c_ | ζ | η | tensormodel |
| --- | --- | --- | --- | --- | --- | --- | --- | --- | --- | --- |
| All residues | 2.63±0.04 | 2.63±0.04 | 2.63±0.04 | - | - | - | 6.34±0.09 | - | - | iso (rej) |
|  | 2.13±0.19 | 2.13±0.19 | 3.62±0.15 | 11±15 | 88±17 | - | 6.34±0.13 | 1.69±0.31 | - | sym (sel) |
|  | 2.02±0.19 | 2.24±0.19 | 3.59±0.20 | 11±5 | 91±6 | 95±35 | 6.37±0.13 | 1.68±0.25 | 0.23±0.26 | aniso (rej) |
| PS (2-7,26-31) | 2.53±0.04 | 2.53±0.04 | 2.53±0.04 | - | - | - | 6.59+-0.09 | - | - | iso (rej) |
|  | 2.21±0.21 | 2.21±0.21 | 3.18±0.22 | 18±22 | ± | - | 6.58±0.11 | 1.44±0.38 | - | sym (sel) |
|  | 2.02±0.15 | 2.41±0.17 | 3.20±0.19 | 14±10 | 86±6 | 11±31 | 6.55±0.09 | 1.44±0.41 | 0.59±0.24 | aniso (rej) |
| calc ^a^ | 1.40 | 2.21 | 5.55 | 2 | 89 | 21 | 5.46 | 3.08 | 0.32 |  |
| calc ^b^ | 1.16 | 1.83 | 4.59 | 2 | 89 | 21 | 6.61 | 3.08 | 0.32 |  |

The diffusion tensors were computed using all or proximal stem (PS) residues ^13^C relaxation data. For both selections the statistical evaluation selects an axially symmetric diffusion tensor (sym) model, the isotropic (iso) or fully anisotropic (aniso) models are rejected. Units are 10^7^ s^-1^ for D_x_, D_y_ and D_z_, ns for τ_c_ and degrees for α, β, and γ angles. The last two lines report the tensors predicted by ELM (38) from the pdb coordinates at a temperature of 34ºC, with ^a^ predicted values with the default parameters of ELM and ^b^ corrected by η_D2O_ /η_H2O_ (=1.21 at 34ºC).
